# Cyclophilins A, B, and C Role in Human T Lymphocytes Upon Inflammatory Conditions

**DOI:** 10.3389/fimmu.2021.609196

**Published:** 2021-03-30

**Authors:** Sandra Gegunde, Amparo Alfonso, Rebeca Alvariño, Eva Alonso, Luis M. Botana

**Affiliations:** ^1^ Departamento de Farmacología, Facultad de Veterinaria, Universidad de Santiago de Compostela, Lugo, Spain; ^2^ Grupo Investigación Biodiscovery, Instituto de Investigación Sanitaria de Santiago de Compostela, Santiago de Compostela, Spain

**Keywords:** cyclophilin, inflammation, cyclophilin A inhibitors, CD147 receptor, T lymphocyte, migration

## Abstract

Cyclophilins (Cyps) are a group of peptidyl-prolyl *cis/trans* isomerases that play crucial roles in regulatory mechanisms of cellular physiology and pathology in several inflammatory conditions. Their receptor, CD147, also participates in the development and progression of the inflammatory response. Nevertheless, the main function of Cyps and their receptor are yet to be deciphered. The release of CypA and the expression of the CD147 receptor in activated T lymphocytes were already described, however, no data are available about other Cyps in these cells. Therefore, in the present work intra and extracellular CypA, B and C levels were measured followed by induced inflammatory conditions. After activation of T lymphocytes by incubation with concanavalin A, both intra and extracellular Cyps levels and the CD147 membrane receptor expression were increased leading to cell migration towards circulating CypA and CypB as chemoattractants. When CypA was modulated by natural and synthetic compounds, the inflammatory cascade was avoided including T cell migration. Our results strengthen the relationship between CypA, B, and C, their receptor, and the inflammatory process in human T lymphocytes, associating CypC with these cells for the first time.

## Introduction

Cyclophilins (Cyps) belong to a subgroup of proteins called immunophilins. This well-studied family is highly conserved in all organisms and possesses peptidyl-prolyl *cis-trans* isomerase (PPIase) activity. Cyps are involved in cell signaling, trafficking, protein folding, and transcription (1). The PPIase activity of Cyps is inhibited by the potent immunosuppressant cyclosporine A (CsA) produced by the fungus *Tolyplocadium inflatum* (2). Furthermore, some Cyps, namely CypA, B and C among others, have been implicated in inflammatory-related diseases (3, 4). Inflammation is a complex process, in its development, immune cells, such as monocytes, macrophages and T lymphocytes are recruited and accumulated into tissues (5). This recruitment and trafficking are key steps during the activation of the inflammatory response. In this process, extracellular CypA act as a chemotactic agent for leukocytes. Upon inflammatory conditions, endothelial cells (ECs), vascular smooth muscle cells, inflammatory lesions, and monocytes generate high amounts of CypA. In this way, CypA is released and begins a chemotactic response for leukocytes (6). Extracellular CypA, at the same time, promotes inflammatory responses, such as ROS production, extracellular signal-regulated kinase (ERK) 1/2 phosphorylation, DNA synthesis, and vascular smooth muscle cell proliferation (7). Furthermore, high levels of CypA are found in synovial fluids from patients with rheumatoid arthritis, in plasma from patients with type 2 diabetes, and serum from patients with sepsis (8, 9). CypB also possesses proinflammatory properties and is secreted by several cells. This immunophilin plays a principal role in ER redox homeostasis. High CypB levels are found in inflammatory processes, like sepsis and rheumatoid arthritis, as well as CypA. Besides, platelet adhesion to collagen and the neutrophil chemotaxis is promoted by CypB (10). Furthermore, CypA and B expression is augmented in the cytosol of T lymphocytes from patients with coronary artery disease (CAD) (11). Less is known about the role of CypC, albeit this immunophilin participates in redox ER homeostasis and has been linked to macrophage activation (12). Moreover, recent studies described high CypC serum levels in patients with acute CAD when compared with controls. In this sense, this immunophilin was proposed as a CAD biomarker (4, 13). CAD is an inflammatory-based pathology, whereby CypA and B, along with CypC also play a relevant role (13–15).

Extracellular CypA and B bind to the CD147 membrane receptor and modulate inflammatory pathways (10, 16). CD147 is a type I transmembrane glycoprotein that belongs to the immunoglobulin superfamily (17). This receptor is overexpressed in activated T and B lymphocytes, neutrophils, monocytes, macrophages, and dendritic cells, participating in the immune and inflammatory response (18). Besides, chemotaxis, phosphorylation of ERK 1/2 and cell signaling are mediated by Cyps through CD147 (19). Moreover, the extracellular interaction of CypA/CD147 is upregulated in inflammatory and autoimmune diseases (11, 18). Consequently, there is a clear interconnection between CypA, CypB, the CD147 receptor, and inflammatory-related diseases. T lymphocytes are critical players in the progression of the inflammatory response. Although the activation of these cells involves several well-known pathways promoting proinflammatory mediators release, Cyps role is still undefined. CD147 receptor expression and CypA release were described in T cells upon inflammatory conditions (6, 20). However, the intracellular expression of this protein as well as CypB or C profile were not before studied in these conditions.

**Figure 1:**
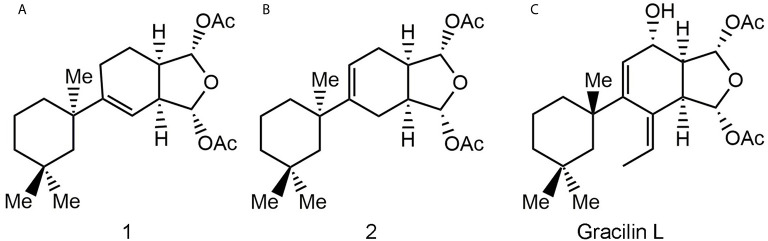
Natural gracilin and two synthetic analogues structures. Compound **1 (A)**, **2 (B)**, and Gracilin L **(C)**.

The modulation of Cyps with specific drugs has been used to better understand the inflammatory response. In this way, recent data have shown that natural gracilins and synthetic gracilin A (GraA)-analogues can bind certain Cyps and inhibit the calcineurin phosphatase activity, in the same way as the well-known CypA inhibitor CsA (20–22). Gracilins, diterpenes derivatives extracted from the sponge *Spongionella gracilis*, have shown neuroprotective, antioxidant, and anti-inflammatory capacities through their binding affinities toward CypA and/or CypD (22–24). Also, these compounds modulate the expression of CD147 on activated T lymphocytes (20, 22). Thus, two synthetic GraA-analogues, bis-acetoxy furanoses, compounds **1** and **2** (**Figure 1**), are inhibitors of CypA (21). They showed kinetic equilibrium dissociation constants (K_D_) values in the nanomolar range as measured by plasmon resonance experiments (**Table 1**) (21, 22). Moreover, these small molecules decrease pro-inflammatory mediators release, inhibit the calcineurin phosphatase activity, target the translocation of pro-inflammatory proteins and promote antioxidative mechanisms through CypA modulation (21, 25). Therefore, they showed interesting anti-inflammatory capacities and are valuable tools to improve knowledge about the role of Cyps in inflammatory conditions.

**Table 1:** Kinetic equilibrium dissociation constant (K_D_) values for binding of *Spongionella* derived compounds and CsA to CypA.

	K_D_ (nM)	Calcineurin inhibition (%)	IL-2 release (%)
**Gracilin L**	24.85 ± 6.83	≈ 10	71.8 ± 11.6
**Compound 1**	5.34 ± 1.68	22.3 ± 6.1	44.5 ± 2.15
**Compound 2**	7.67 ± 1.61	31.0 ± 3.4	36.1 ± 5.9
**Cyclosporin A**	6.83 ± 1.1	17.3 ± 4.5	66.75 ± 8.25

Inhibition of calcineurin phosphatase activity expressed in percentage of control cells. IL-2 inhibition is expressed in the percentage of control cells. For calcineurin and IL-2 experiments, cells were treated with GraL (1 μM), compounds **1**, **2** (0.1 µM), or CsA (0.2 µM) for 2 h and then were stimulated with Con A (50 μg/mL) for 48 h.

In this context, in the present study CypA, B and C levels and their association with the CD147 receptor upon inflammatory conditions are studied in human T lymphocytes as well as their modulation with natural and synthetic gracilins.

## Material And Methods

### Chemicals and Solutions

The natural compound Gracilin L was kindly donated by Dr. Marcel Jaspars from the University of Aberdeen, Scotland, UK. The synthetic compounds, **1** and **2**, were kindly donated by Dr. Romo group from the Department of Chemistry and Biochemistry, Baylor University, Texas, US. The sponge-derived GraA was used to obtain bioactive small molecules, compound **1** and **2**, through pharmacophore-directed retrosynthesis (PDR) as described previously (21).

Human Cyclophilin A ELISA kit (CSB-E09920H), Human Cyclophilin B ELISA kit (CSB-E11218H) and Human Peptidyl-Prolyl *cis-trans* Isomerase C (PPIC) Elisa kit (CSB-EL018473HU) were purchased from Cusabio (Madrid, Spain). FITC anti-human CD147 was from Immunostep (Salamanca, Spain). Percoll was obtained from GE Healthcare (Madrid, Spain). The human Pan T cell Isolation Kit and the monoclonal antibody to human CD3, clone BW264/56, FITC were purchased from Miltenyi Biotec (Germany). The composition of phosphate buffer saline (PBS) solution used for human T lymphocytes purification was (in millimolar) 137 ClNa, 8.2 Na_2_HPO_4_, 3.2 KCl, 1.5 KH_2_PO_4_ and 2 EDTA, pH 7.2-7.4. The composition of Umbreit saline solution was (in millimolar) 119 NaCl, 5.94 KCl, 22.85 NaHCO_3_, 1.2 MgSO_4_, 1.2 NaH_2_PO_4_, and 1 CaCl_2_. Glucose 1 g/L was added to the medium. Roswell Park Memorial Institute (RPMI) medium, Foetal bovine serum (FBS), penicillin/streptomycin, SuperSignal west pico, SuperSignal west femto, anti-Actin monoclonal antibody (cat. No. #ACTN05 (C4), lot. #SK2474691D), anti-CypC antibody (cat. No. #PA5-69299, lot. #TD2552443D) were obtained from Thermo Fisher Scientific (Madrid, Spain). Anti-PPIA polyclonal antibody (cat. No. E-AB-15306, lot. DK0674), anti-CypB antibody (cat. E-AB-22123, lot. AC0235), were bought in Elasbscience (Madrid, Spain). Polyacrylamide gels and molecular weight marker Precision Plus Protein Standards Kaleidoscope were obtained from Bio-Rad (Barcelona, Spain). Protease Inhibitor Complete Tablets and Phosphatase Inhibitor Cocktail Tablets were from Roche (Madrid, Spain). Cyclosporine A (purity ≥98.5%), Chemotaxis 5 μm 96-Well Cell Migration Assays (ECM512), Percoll (GE17-0891-01), Polyvinylidene difluoride (PVDF) membrane, Bovine Serum Albumin (BSA), endoplasmic reticulum isolation kit (cat# ER0100), and the rest of chemicals and reagents were obtained from Sigma-Aldrich (Madrid, Spain). Recombinant human cyclophilin A protein (active) (ab86219) was purchase from Abcam (Germany). Recombinant human cyclophilin B protein (active) (Cat. No. ABIN1304473) was bought in Antibodies-online. Stock solutions of drugs were done in dimethyl sulfoxide (DMSO). The amount of DMSO added to the cells was always lower than 0.01%. The same amount of vehicle is added to control cells.

### Human T Lymphocytes Isolation

Volunteers without known inflammatory-related disease or other conditions, were enrolled in the present study. This study was reviewed and approved by the institutional and regional ethical boards according to the Declaration of Helsinki (Reference: 2016/508, Approved date: December 19, 2016). All participants have given their written informed consent before the study began. Peripheral blood for T lymphocytes isolation was obtained from each subject using tubes with EDTA (BD vacutainer blood collection tubes). The human T lymphocytes were isolated as described before (11). Briefly, 3 mL of isotonic percoll (57.5%) were placed at the bottom of a 10 mL tube, then 4 mL of diluted blood was added to the surface of the isotonic percoll. The tubes were centrifuged at 3000 rpm for 25 min. T lymphocytes appearing in the interface were rinsed with PBS-EDTA and centrifuged at 1500 rpm for 10 min. Pan T cell Isolation kit was used to purify T cells from this population according to the manufacturer’s instructions. The purity of T lymphocytes obtained was ≥ 98%. The cell purity was measured by flow cytometry, using a monoclonal antibody to human CD3 labeled with FITC. The population of T cells obtained from one subject was used for a single replica (biological replica) and was measured by triplicate or duplicate (technical replica). Therefore, T cells from three different donors were used in each experiment.

### T Lymphocytes Culture and Activation

Purified T lymphocytes from control subjects were maintained in RPMI supplemented with 10% FBS, penicillin (100 U/mL), and 100 μg/mL streptomycin at 37°C in a humidified atmosphere of 5% CO_2_ and 95% air. 2 x 10^6^ human T lymphocytes were seeded in a 48-well plate in a volume of 400 μL. Cells were pre-treated with GraL (1 μM), compound **1**, compound **2** (0.1 μM) or CsA (0.2 μM) for 2 hours. Then, these cells were activated with Con A (50 μg/mL) for 48 hours and then were used for experiments of ELISA, westerns blot and flow cytometry (20, 21). In each experiment unstimulated cells are used as control. These cells were incubated with the same amount of vehicle than treated cells.

### Measurement of Extracellular Cyclophilin A, B, and C Levels

Levels of CypA, B, and C were measured in a culture medium obtained from T lymphocytes. After compounds pre-treatment and Con A activation cells were centrifuged at 2000 rpm, at 4°C for 5 min. Cyps measurements were done in the media collected, using ELISA kits following the manufacturer’s instructions as previously described (4). The optical density of each well was obtained using a spectrophotometer microplate reader (Sinergy 4 de Biotek). All samples were run in duplicate, and the concentrations were calculated using a standard curve. The intra and inter-assay coefficients of variation of the ELISA kits were <10%. No cross-reactivity was observed between Cyp antibodies.

### CD147 Receptor Expression in T Lymphocytes

The CD147 receptor expression was assessed in treated cells as described before (20). In brief, 1 x 10^6^ washed T cells were suspended in 50 μL of PBS/BSA and were incubated with 20 μL of FITC Anti-Human CD147 for 90 min in darkness at 4°C and 300 rpm. After that, cells were washed and resuspended in 100 μL of PBS with 1% of paraformaldehyde, then were analyzed by flow cytometry technique. The IDEAS ImageStream Analysis software was used to interpret the data obtained from the measurements of the fluorescence intensity.

### ER and Golgi Apparatus Isolation

Isolation of the ER and Golgi apparatus was performed in T lymphocytes with a commercial kit (Sigma endoplasmic reticulum isolation kit) according to the manufacturer’s instructions. In brief, cells were suspended in a hypotonic extraction buffer. After 20 min incubation on ice, cells were centrifuged at 600 g for 5 min and 4°C, and the supernatant was discarded. Then isotonic extraction buffer was added, and the homogenates were centrifuged at 1,000 x g for 10 min and 4°C. The supernatant was subjected to a series of centrifugation steps and then the microsomal fraction containing the ER and Golgi apparatus was obtained and used for western blot analysis.

### Western Blot Analysis

**Figure 2:**
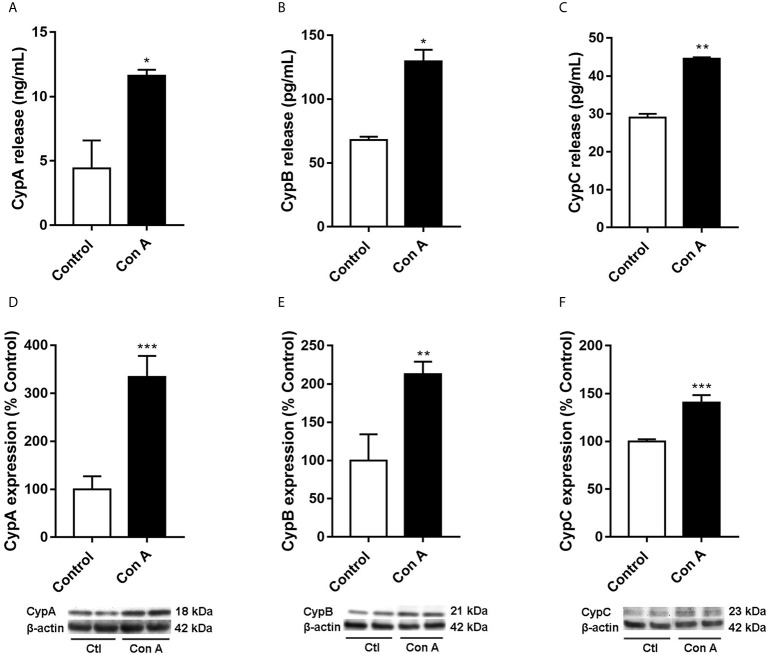
Intra and extracellular CypA, B, and C levels in human T lymphocytes stimulated with Con A Cells were treated with Con A (50 μg/mL) for 48 h. CypA **(A)**, CypB **(B)**, and CypC **(C)** levels were measured by ELISA in the medium of T lymphocytes. The intracellular expression of CypA **(D)**, CypB **(E)**, and CypC **(F)** was measured by western blot in cytosolic lysates of T lymphocytes. Band intensity was normalized by β-actin. Data are the result of average ± SEM (n = 3). The values are shown as the difference between cells treated with Con A and untreated cells, *p < 0.05, *p < 0.01 and ***p < 0.001. One-way ANOVA test with Dunnet’s *post hoc* analysis and Student´s t-test were used for statistical analysis.

**Figure 3:**
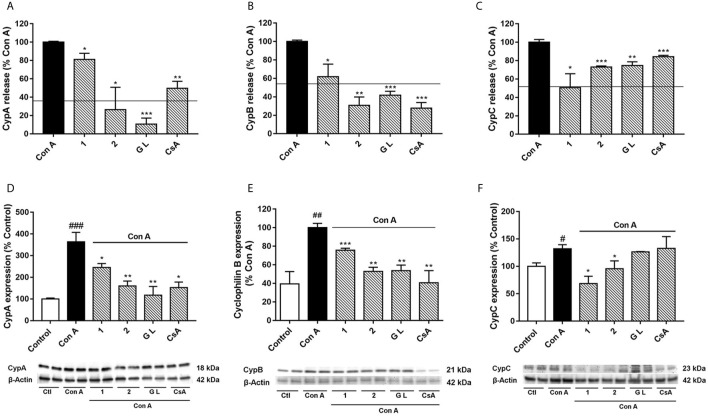
Effect of compounds **1**, **2**, and GraL over intra and extracellular CypA, B and C levels in human T lymphocytes stimulated with Con A Cells were pre-treated with compound **1**, **2** (0.1 µM), GraL (1 μM) or CsA (0.2 µM) for 2 h and then were stimulated with Con A (50 μg/mL) for 48 h. CypA **(A)**, CypB **(B)**, and CypC **(C)** levels were measured by ELISA in the medium of T lymphocytes. The line represents the unstimulated cells values. The intracellular expression of CypA **(D)**, CypB **(E)**, and CypC **(F)** was measured by western blot in cytosolic lysates of T lymphocytes. Band intensity was normalized by β-actin. Data are the result of average ± SEM (n = 3). The values are shown as the difference between cells treated with Con A and cells treated with compounds plus Con A, *p < 0.05, **p < 0.01 and ***p < 0.001. Values of cells treated with Con A were compared with control cells, ^#^ *p* < 0.05, ^##^ *p* < 0.01 and ^###^ *p* < 0.001. One-way ANOVA test with Dunnet’s *post hoc* analysis and Student´s t-test were used for statistical analysis.

Western blot was performed to determine the expression of CypA and CypB in the cytosol of T cells as previously described and as well as the expression of CypC in the Golgi apparatus and ER (25). In brief, to extract the cytosolic proteins, the pellet was suspended for 15 min in 100 μL of ice-cold hypotonic lysis, then 5 μL of Triton X-100 was added and centrifuged for 10 min at 4°C and 3000 rpm. The supernatant was collected as the cytosolic fraction. Protein cytosol concentration was determined by Direct Detect (Millipore). CypC was studied in ER and Golgi-apparatus as described above. 10 μg of total protein of cell lysates were resolved on a 4-20% polyacrylamide gel and transferred onto PVDF membrane for electrophoresis. To determine the protein size Precision Plus Protein Standards Kaleidoscope molecular weight marker was used. Afterward, membranes were blocked with 0.5% BSA diluted in wash buffer and antibody incubation was performed using SNAP i.d. protein detection system. The immunoreactive bands were analyzed using the Supersignal West Pico or Supersignal West Femto and the Diversity GeneSnap software (Syngene). CypA was detected with anti-PPIA (1:2000), CypB was measured with anti-CypB (1:2000) and CypC was analyzed with anti-CypC antibody (1:1000). The signal was normalized by that of β-actin (1:2000). Cross-reactivity between Cyp antibodies was checked with pure Cyps and no interactions were observed (see **Supplementary Material**, **Figures S1A–C**). Uncropped images of western blot experiments showed in **Figures 2D–F** and **3D–F** are showed in supplementary material (**Figures S2–S5**).

### Chemotaxis Assay

Purified human T lymphocytes were used to carry out the chemotaxis cell migration experiments using the Chemotaxis 5 μm 96-Well Cell Migration Assay as described before (20). In this commercial kit, the number of migratory cells is quantified by using a fluorescent CyQuant GR Dye. The assay was performed following the manufacturer’s instructions. Briefly, 150 μL of serum-free RPMI culture medium in the presence or absence of chemoattractant (50 ng/mL CypA or 5 ng/mL CypB) were added to the 96-well feeder tray plate. Then, untreated, or treated cells (200.000 per condition and 100 μL of culture media without FBS) were placed on the insert membrane for 24 h at 37°C and 5% CO2. After 24 hours, migratory cells were incubated for 15 min at room temperature with Lysis buffer containing the CyQuant GR Dye. Afterward, the fluorescence of lysed T lymphocytes was measured in a spectrophotometer plate reader at 480 nm excitation and 520 nm emission.

### Statistical Analysis

Results were expressed as mean ± SEM of a minimum of three independent experiments (three biological replicates) and were performed by duplicate or triplicate (technical replicates). Comparisons were analyzed using ANOVA statistical analysis followed by *post hoc* Dunnet’s *t*-test. *P* values < 0.05 were considered statistically significant.

## Results

Upon inflammatory conditions, CypA secretion from T lymphocytes was previously described (6, 20, 26). Nevertheless, no data are available regarding intracellular CypA expression or CypB and C levels in these conditions. So, our initial premise was to check Cyps profile in Con A-stimulated T cells. Purified human T lymphocytes were activated with Con A, a known inflammatory stimulator, for 48 hours, and intra and extracellular CypA levels were measured. As was expected **Figure 2A**, the release of CypA was more than two times higher in cells treated with Con A compared with untreated cells (*p* < 0.05). Moreover, as **Figure 2D** shows, in these conditions, the intracellular CypA expression was also raised (*p* < 0.001), more than three times. Then, CypB and C were measured in activated T lymphocytes. As **Figure 2B** shows, the release to the medium of CypB was twice higher on Con A-treated cells. Besides, the intracellular CypB expression was significantly increased in stimulated T cells comparing with untreated cells (**Figure 2E**, *p* < 0.01). As happened with CypA and B, the liberation of CypC was also significantly elevated (**Figure 2C**). The CypC levels were significantly increased in the activated T lymphocyte’s medium than in untreated cells (*p* < 0.01). Also, as **Figure 2F** shows, the intracellular expression of CypC was augmented, 40%, in cells treated with Con A (*p* < 0.05). Therefore, in addition to CypA and B, upon inflammatory conditions, CypC is also produced and released by activated T lymphocytes.

To better understand the relationships between CypA, B, and C under inflammatory conditions, CypA selective inhibitors were used in activated T cells, and Cyps profiles were checked again. For this purpose, the natural GraL, two synthetic GraA-analogues, compounds **1** and **2**, and CsA were tested in Con A-stimulated human T lymphocytes, then, Cyps release (**Figures 3A–C**) and their intracellular expression (**Figures 3D–F**) were measured. In the present study, we have focused on these compounds because as **Table 1** shows, they have shown high affinities by CypA (nM range of K_D_), inhibition of calcineurin phosphatase activity (10-30%) and inhibition of interleukin (IL)-2 release in the same level as CsA (21, 22). Moreover, these synthetic compounds have interesting anti-inflammatory and neuroprotective activities in the same way as natural gracilins (20–22, 24, 27). In the following experiments, the concentration of 1 μM of GraL, 0.1 μM of compounds **1** and **2**, and 0.2 μM of CsA were used. Since these concentrations have shown the greatest anti-inflammatory activity without compromising cell viability in previous experiments, both in T lymphocytes and glial cells (20, 21, 25). As shown in **Figure 3A**, the liberation of CypA was significantly reduced, when T cells were preincubated with compound **1** (*p* < 0.05), compound **2** (*p* < 0.05) or GraL (*p* < 0.001). The CypA liberation was also inhibited, by CsA, as it was already described (20). As **Figure 3B** shows, the CypB levels were highly reduced in the medium of stimulated T cells pre-treated with compound **1** (*p* < 0.05), **2**, GraL and CsA (*p* < 0.01). Furthermore, in **Figure 3C**, CypC release from T lymphocytes was also significantly decreased by natural GraL and the two synthetic compounds (*p* < 0.05). CsA significantly modifies CypC release, with the lower effect. Therefore, when CypA is modulated in activated T cells, extracellular CypB and C levels are restored near to non-inflammatory conditions. Next, intracellular CypA, B and C expression were also tested. As **Figure 3D** shows, CypA expression was reduced in activated T cells by compounds **1**, **2**, or GraL pre-treatments, reaching unstimulated cell levels (p < 0.05). Intracellular CypA expression was also reduced by CsA (*p* < 0.05). Furthermore, the intracellular CypB expression was significantly reduced to control values after pre-treatment of Con A-stimulated T cells with Spongionella derivatives, or CsA (**Figure 3E**, *p* < 0.01). Moreover, it is important to highlight that intracellular CypA and B levels follow the same profile (**Figures 3D, E**). Finally, as **Figure 3F** shows, compounds **1** and **2** significantly diminished CypC intracellular expression in activated T lymphocytes (*p* < 0.01, *p* < 0.05), while the pre-treatment with GraL or CsA did not modify intracellular CypC levels. Thus, the modulation of CypA levels with Spongionella-derived compounds substantially affects CypA, B, and C profiles. These compounds are more potent than the classical immunosuppressive CsA.

**Figure 4:**
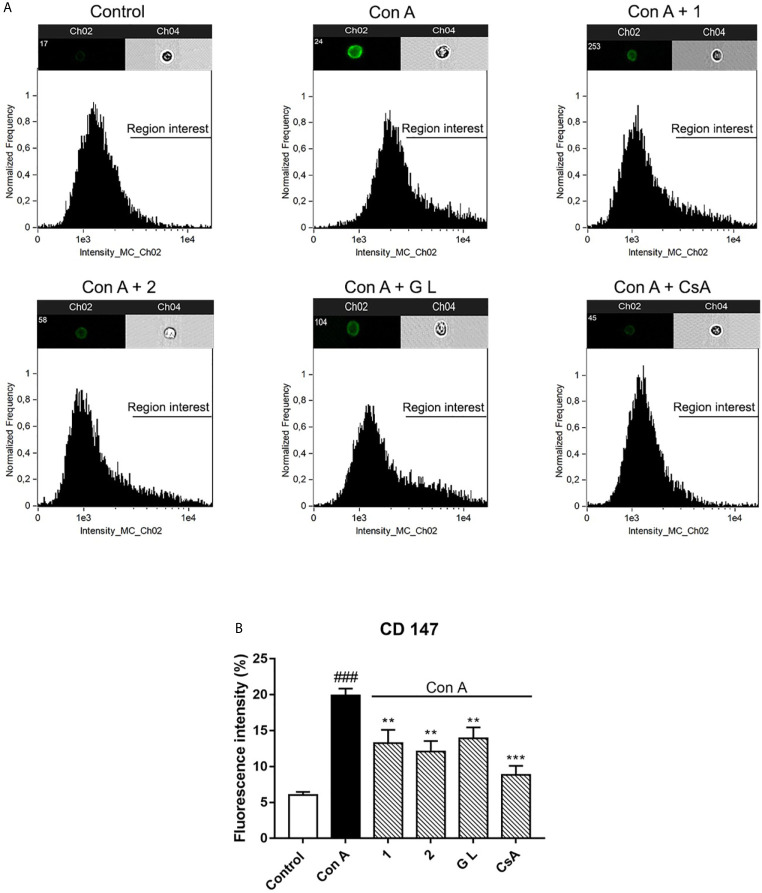
Effect of compounds **1**, **2**, and GraL over plasma membrane levels of CD147 receptor in human T lymphocytes stimulated with Con A Cells were pre-treated with compound **1**, **2** (0.1 µM), GraL (1 μM) or CsA (0.2 µM) for 2 h and then were stimulated with Con A (50 μg/mL) for 48 h. Representative cellular images of the expression of plasma membrane CD147 receptor: brightfield images are shown in channel 4 and FITC intensity images are presented on channel 2 **(A)**. Histogram of the expression of plasma membrane CD147 receptor **(B)**. Data are the result of average ± SEM (n = 3). The values are shown as the difference between cells treated only with Con A and compared to cells treated with compounds plus Con A, **p < 0.01 and ***p < 0.001. Values of cells treated with Con A were compared with control cells, ^###^ *p* < 0.001. One-way ANOVA test with Dunnet’s *post hoc* analysis and Student´s t-test were used for statistical analysis.

CD147 is the extracellular receptor for CypA and B and is overexpressed in several inflammatory pathologies. Moreover, the membrane expression of this receptor is controlled by these Cyps (10, 28, 29). Hence, our next step was to check if CypA modulation by natural and synthetic compounds affects receptor expression. As **Figure 4** shows, the CD147 receptor is at least 20% expressed in T cell surface after Con A stimulation *versus* 6% in the surface of untreated cells. That is, the receptor was three times more present on the cell surface of stimulated cells than in untreated cells (*p* < 0.01). This increase in the membrane receptor expression on activated T cells is in line with the results described before (20). However, the receptor expression was halved in the presence of compounds **1**, **2**, and GraL (*p* < 0.01). Also, the expression of CD147 in the membrane of activated T lymphocytes was significantly reduced after CsA treatments (*p* < 0.01). Therefore, CypA modulation decreased the CD147 expression on activated T lymphocytes.

**Figure 5:**
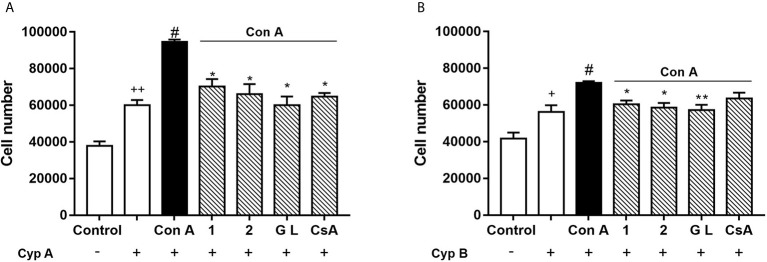
Effect of compounds **1**, **2**, and GraL over human T lymphocytes migration. Cells were pre-treated with compound **1**, **2** (0.1 µM), GraL (1 μM) or CsA (0.2 µM) for 2 h and then were stimulated with Con A (50 μg/mL) for 48 h. Then, T cells were placed in the migration chamber for 24 hours in the presence of the chemoattractant CypA (50 ng/mL) **(A)** or CypB (5 ng/mL) **(B)**. Controls of untreated T cells in the presence or absence of Cyps are also included. Data are the result of average ± SEM (n = 3). The values are shown as the difference between Con A migratory cell numbers compared to cells treated with compounds plus Con A, *p < 0.05 and **p < 0.01. Values of cells treated with Con A were compared with cells in the presence of CypA or B, ^#^ *p* < 0.05. Values of untreated cells in absence of CypA or B were compared with untreated cells in presence of CypA and B, ^+^ *p* < 0.05 or ^++^ *p* < 0.01. One-way ANOVA test with Dunnet’s *post hoc* analysis and Student´s t-test were used for statistical analysis.

Extracellular CypA and B act as chemoattractants for T cells through their interactions with the CD147 membrane receptor (3, 10, 30). As we previously described, Con A-activated human T lymphocytes can migrate towards CypA, and this effect was blocked by natural gracilins or CsA treatment (20). However, the role of CypB in this cellular model of inflammation as well as the effect of selective CypA modulation is unknown. Therefore, our final step was to check T cell migration under these conditions performing a chemotaxis assay. After 48 hours of Con A-treatment, cells were placed in the insert membrane of the migration chamber, while in the feeder tray CypA or B were used as chemoattracting agents. After 24 hours the number of cells migrated was measured. As **Figures 5A**, **B** show, in control conditions (non-stimulated cells and absence of chemoattractants) around 40,000 cells freely migrate from the upper to the bottom chamber. When CypA (50 ng/mL) was added to the bottom chamber, Figure 8a , 60,020 ± 2,846 cells were attracted. In those conditions, as expected, cell migration was increased by 40% (94,638 ± 1,230 cells) when T cells were previously activated with Con A. If CypA pathway was inhibited by pre-treatment with compound **1**, **2**, GraL, or CsA, the migration promoted by Con A was significantly avoided (*p* < 0.05). Then, CypB (5 ng/mL) was added to the bottom of the chamber to check its efficiency as a T cell chemoattractant. We used this amount because extracellular CypB levels are usually 10 times lower than CypA in inflammatory conditions (4, 13, 31). As **Figure 5B** shows, when CypB was present, 56,229 ± 3,622 of untreated cells migrated to the bottom chamber. When T cells were Con A-stimulated, the migratory cell number was increased (72,049 ± 0,896; *p* < 0.05). In these conditions, T cell migration was avoided when CypA was significantly inhibited by *Spongionella* compounds pre-treatments (*p* < 0.05). In summary, the inhibition of CypA prevents the chemotaxis of activated T lymphocytes, although in presence of high extracellular levels of CypA or B.

## Discussion

In the present study, activated human T lymphocytes were used. It is known that Con A, a plant lectin, has proinflammatory properties and through the antigen receptor activates T lymphocytes. Moreover, Con A stimulates the expression and release of CypA, as well as the migration of T cells (20, 26). Furthermore, the release of CypB and C and their intracellular levels were also increased in Con A-activated T cells, as described for the first time in this work. Therefore, in addition to the well-known CypA, CypB and C are also released by activated T lymphocytes and play an important role in the inflammatory process, either directly or indirectly. It is important to point out that, while intracellular CypA and B are present in the cytosol, intracellular CypC has a different localization, since lysates of microsomal fractions containing the ER and Golgi apparatus were necessary to detect this immunophilin. CypC has been associated with the ER in kidney and human hepatoma cells (32–34). Therefore, from our findings, CypC is linked with the ER in human T lymphocytes whereas CypA and B with the cytosol, and all three are released to the extracellular media from these cells upon inflammatory conditions.

As it was mentioned before, CypA seems to play a key role in inflammatory processes, therefore, it was proposed as an appropriate goal in these pathologies. In this sense, CsA was used in several *in vitro* studies to modulate CypA (35). The immunosuppressive properties of CsA are due to the formation of a ternary complex with CypA and calcineurin. This complex blocks the activation of the nuclear factor of activated T cells (NFAT) calcineurin-dependent, causing the impossibility of T cell of reacting upon sternal stimuli (36). Besides, the inhibition of T cell activation avoids the following cytokine production (2). Nevertheless, CsA causes toxicity over the pancreas, central nervous system, and kidney and it has been related to hypertension and diabetes (37, 38). Moreover, when human embryonic kidney and human hepatoma cell lines were treated with CsA, the release of CypA, B and C were augmented (27, 39). Natural gracilins showed anti-inflammatory capacities by their affinity to CypA (22). Besides, they can modify transcriptional factors and reduce IL-2 release in activated T cells by blocking the activity of calcineurin phosphatase and inhibit CypA release (20, 22, 27). Moreover, it was recently reported that synthetic GraA-derivatives reduce the release of inflammatory mediators, avoid the translocation to the nucleus of inflammatory target proteins and promote antioxidative mechanisms in activated BV2 cells, likely through the CypA modulation (25). In the present paper, GraL and two GraA-derivatives, compounds **1** and **2**, with high CypA affinity, in the CsA range, were chosen. These compounds reduce CypA release and the expression of CypA and CD147 receptors in T cells. CypA reduction data reinforce the specific affinity of compounds over this protein described earlier (21, 25). However, although they show similar CypA affinities, they have different effects on inflammatory hallmarks related to CypA (IL-2 release and calcineurin activity). This could explain the diverse inhibition observed in CypA release.

On the other hand, the expression of CD147 surface membrane receptor is upregulated by inflammation and by the modulation of intracellular CypA (20, 40). In this sense, a CypA binding site located in the transmembrane domain at the residue Pro211 of CD147 is responsible for the receptor transport to the surface. The transport and the expression of the surface membrane receptor were greatly reduced after CsA treatments (41). Therefore, the decrease of CD147 receptor expression observed in the presence of *Spongionella*-derived compounds could be mediated by direct interaction with intracellular CypA (21, 25). Recent data demonstrate that proinflammatory neutrophils, monocytes and leukocytes, and activated T cells when they were treated with an anti-CD147 monoclonal antibody, missed their capability to migrate in the presence of CypA. Furthermore, it has been reported successful treatments for inflammatory processes are based on the blockage of CD147 activity (42). In addition, the decrease in the expression of the surface membrane receptor-mediated by the depletion of intracellular CypA is also important in the reduction of T cell migration observed by these compounds. It should also be noted that if compounds can prevent T lymphocyte migration, they could also prevent neutrophil and monocytes migration, critical cells in the inflammatory response.

Besides the intracellular relevance of CypA in protein folding, trafficking, and signaling functions, extracellular CypA has also an important role. Extracellular CypA is a pro-inflammatory cytokine that fosters inflammation in several cells. Not only promotes leukocyte migration but stimulates the release of IL-1β, IL-6, and IL-8, among others, in macrophages and monocytes (43). The proliferation and migration of vascular smooth muscle cells, as well as the matrix metalloproteinase activation, is promoted by extracellular CypA. This immunophilin also induces the apoptosis of endothelial cells, the expression of adhesion molecules, and stimulates the platelet adhesion and the atheromatous plaque formation in atherosclerosis (6, 28). Furthermore, it has been demonstrated that in several cell types, the activation of NF-κB, ERK1/2, JNK, Akt, and p-38 MAPK are stimulated by extracellular CypA (6, 28). In addition, extracellular CypB seems to have an important function as an intercellular mediator in inflammation and enhances the adhesion of platelets to collagen (44). Nevertheless, no data are available about the functions of extracellular CypC in humans. Therefore, the inhibition of CypA and B released by the *Spongionella*-derived compounds could prevent inflammatory responses in several cell types, in addition to T cells. In this sense, extracellular CypA and B are also interesting targets in the modulation of chronic inflammatory diseases.

As aforementioned, CD147 is the receptor for extracellular CypA and B, and their interactions are needed for cell response. The extracellular receptor for CypC remains unidentified, nevertheless, a binding site for CypC in the CD147 receptor has been hypothesized (19). Therefore, the inhibition in the membrane expression of this receptor could be the indirect cause of the CypB and C reduction induced by *Spongionella*-derived compounds. Although a direct effect over these immunophilins cannot be discarded as well as the inhibition of their PPIase activity. In any case, due to the reduction of intracellular Cyps and/or CD147 receptor, the migration of T cells and therefore, the infiltration of them into the inflammatory site is blocked in the presence of compounds. Consequently, these compounds were able to act over the three Cyps associated with inflammatory-related diseases, restoring Cyps levels up to control values. In this way, the inflammatory pathway activated in some pathologies might be avoided (45). Moreover, extracellular CypA and B act as chemoattractant molecules. Therefore, the inhibition of T cell migration in the presence of CypA and B by compounds could block the subsequent inflammatory cascade (19). It should be also taken into consideration that both CypA and B, in the present study, stimulate the migration of resting T lymphocytes. Hence, by inhibiting these stimuli, the migration and infiltration of leukocytes could also be prevented in non-inflammatory conditions. It is important to emphasize that CypC levels are also reduced by *Spongionella*-derived compounds. This protein is highly augmented in CAD, an inflammatory-related condition, therefore, its direct or indirect modulation could be a useful therapeutic strategy in inflammatory-based pathologies (4, 11, 13). Moreover, the decrease in CypC expression and release are more evident in the presence of compound **1** than compound **2** or GraL. This discrepancy may point to different cell permeability, other unknown cellular targets, or variances in their chemical structure. Compound **1** is a regioisomer of **2**, that is, it has a double bond in the cyclohexene ring in a different position than compound **2** and GraL (20, 21, 25). For these reasons, these compounds are proposed in the present study as useful probe molecules to directly modulate CypA and its receptor and to avoid the T lymphocyte migration.

Furthermore, in human activated T lymphocytes, the release and intracellular expression of CypA, B, and C, as well as the expression of the CD147 membrane receptor is upregulated and can be modulated with known inhibitors. Extracellular CypA and B are chemoattractants for activated T cells when these proteins are increased within the cell.

## Data Availability Statement

The original contributions presented in the study are included in the article/supplementary material. Further inquiries can be directed to the corresponding author.

## Ethics Statement

The studies involving human participants were reviewed and approved by Comité de ÉticA de la Investigación de Santiago-Lugo acording to the Declaration of Helsinki (Reference: 2016/508, Approved date: December 19, 2016). The patients/participants provided their written informed consent to participate in this study.

## Author Contributions

Conceptualization: SG and AA. Methodology and formal analysis: SG and RA. Validation: EA. Writing-Original Draft Preparation: SG. Writing-Review and Editing: AA, LB. Supervision: AA. Funding’s acquisitions: AA and LB. All authors contributed to the article and approved the submitted version.

## Funding

The research leading to these results has received funding from the following FEDER cofunded-grants. From Consellería de Cultura, Educación e Ordenación Universitaria, Xunta de Galicia, Spain, 2017 GRC GI-1682 (ED431C 2017/01). From CDTI and Technological Funds, supported by Ministerio de Economía, Industria y Competitividad, Spain, ISCIII/PI19/01248, ISCIII/PI19/00879. In European Union, Interreg AlertoxNet EAPA-317-2016, Interreg Agritox EAPA-998-2018, and H2020 778069-EMERTOX. SG was supported by a fellowship from FIDIS, Spain.

## Conflict of Interest

The authors declare that the research was conducted in the absence of any commercial or financial relationships that could be construed as a potential conflict of interest.
