# Supplementary material for: Cyclophilins A, B, and C Role in Human T Lymphocytes Upon Inflammatory Conditions
Source: Front Immunol. 2021 Mar 30;12:609196. doi: 10.3389/fimmu.2021.609196 (PMC8042163; doi:10.3389/fimmu.2021.609196)
Supplement: Supplementary file 1 [file DataSheet_1.pdf]

# Supplementary figure S1

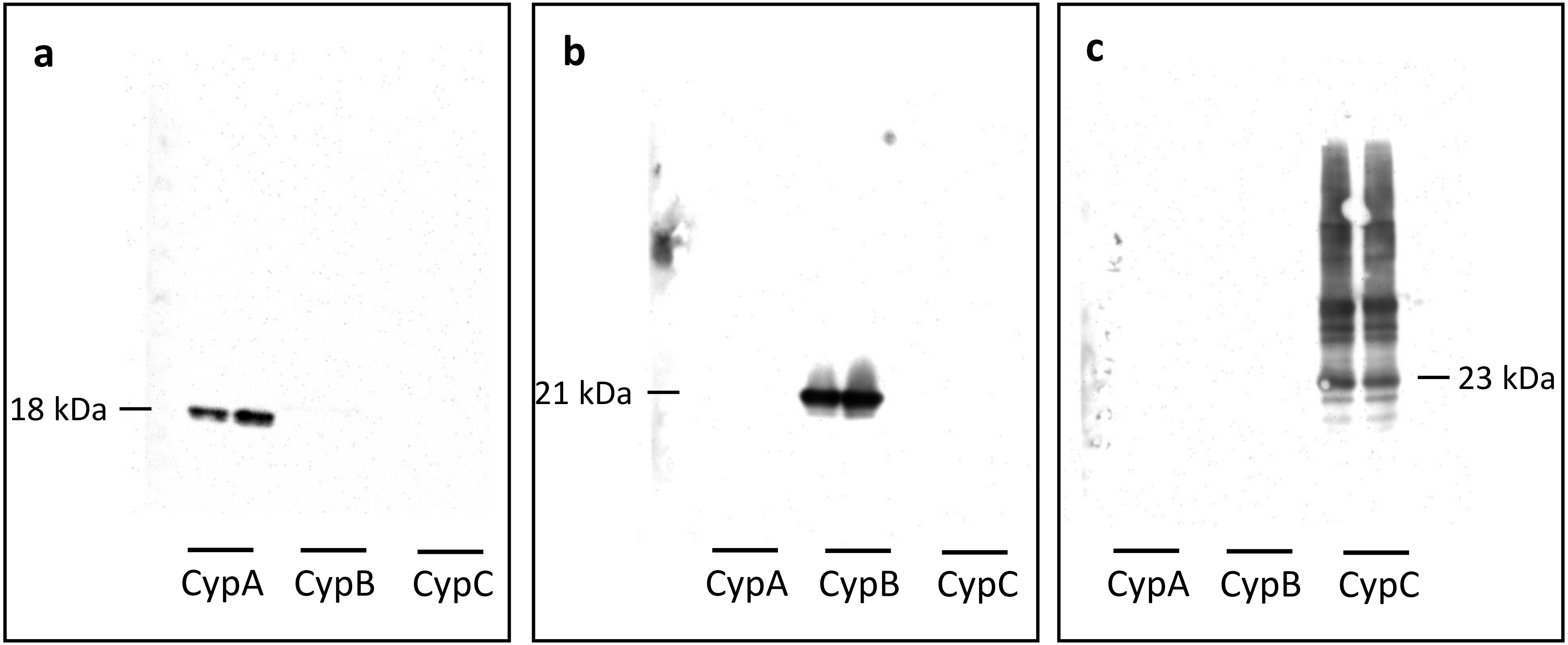


**Figure S1a**. Images of western blot using anti-CypA antibody. The immunoreactive bands were analysed using the Supersignal West Pico.

**Figure S1b**. Images of western blot using anti-CypB antibody. The immunoreactive bands were analysed using the Supersignal West Pico.

**Figure S1c**. Images of western blot using anti-CypC antibody. The immunoreactive bands were analysed using the Supersignal West Pico.

# Supplementary figure S2

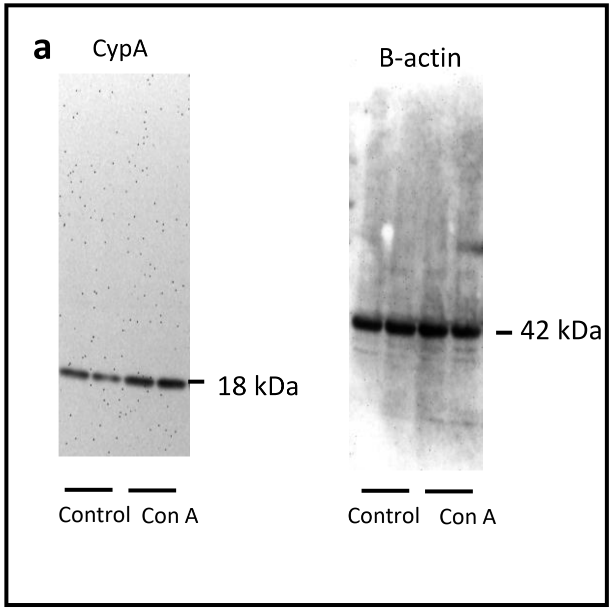


**Figure S2a**. Uncropped images of western blot for CypA used in figure 2d. The immunoreactive bands were analysed using the Supersignal West Pico.

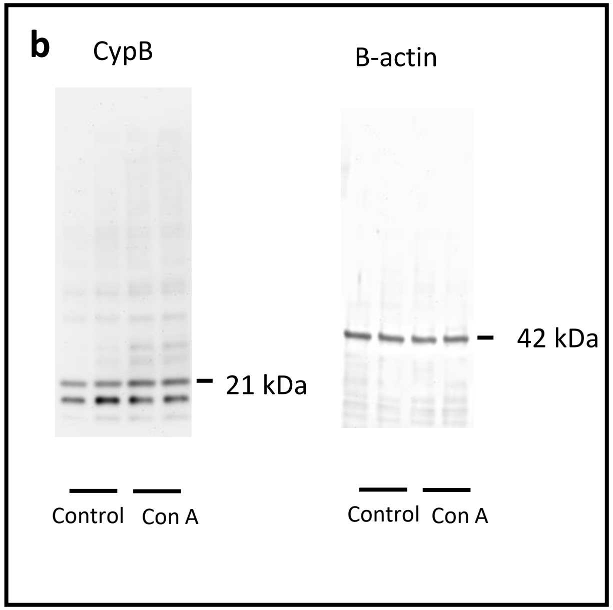


**Figure S2b**. Uncropped images of western blot for CypB used figure 2e. The immunoreactive bands were analysed using the Supersignal West Pico.

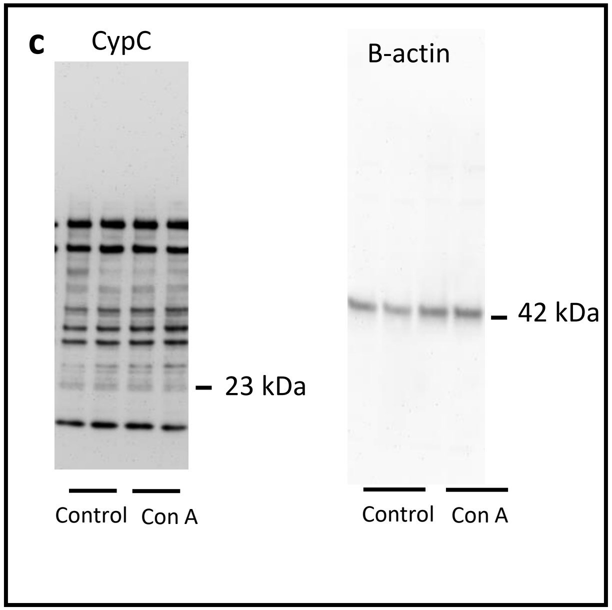


**Figure S2c**. Uncropped images of western blot for CypC used in figure 2f. The immunoreactive bands were analysed using the Supersignal West Femto.

# Supplementary figure S3

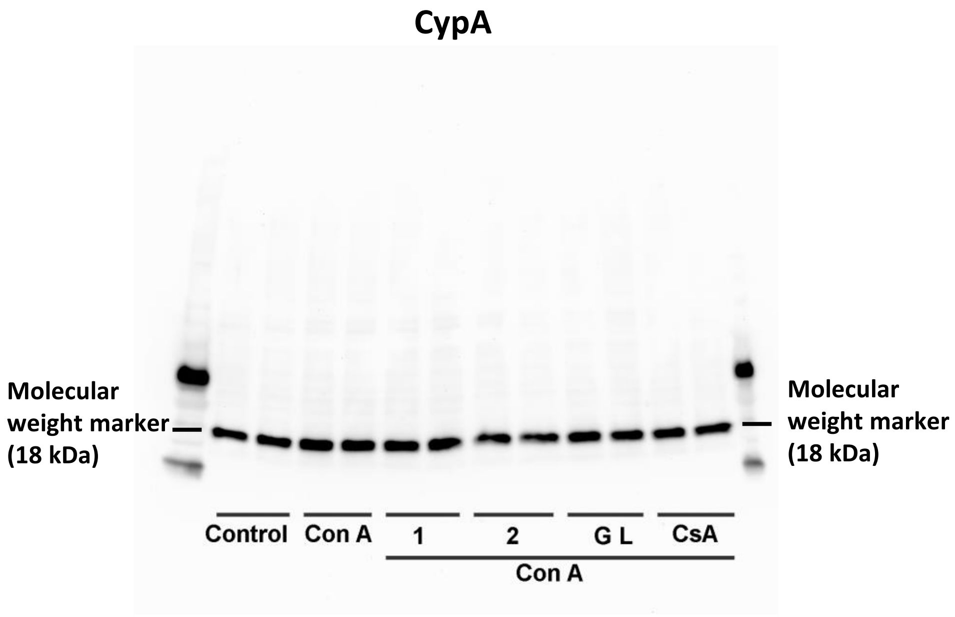


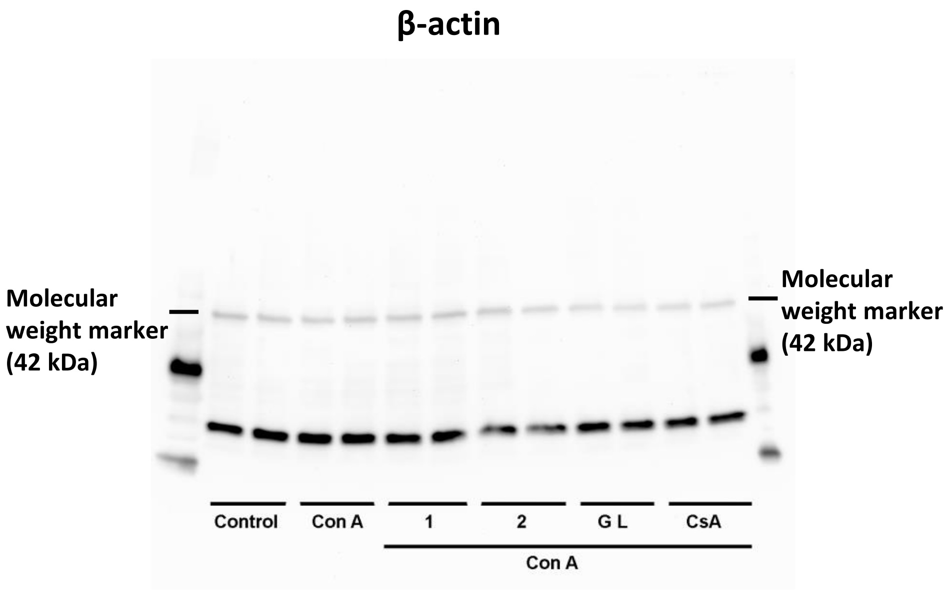


**Figure S3**. Uncropped images of western blot for CypA used in figure 3d. The immunoreactive bands were analysed using the Supersignal West Pico.

# Supplementary figure S4

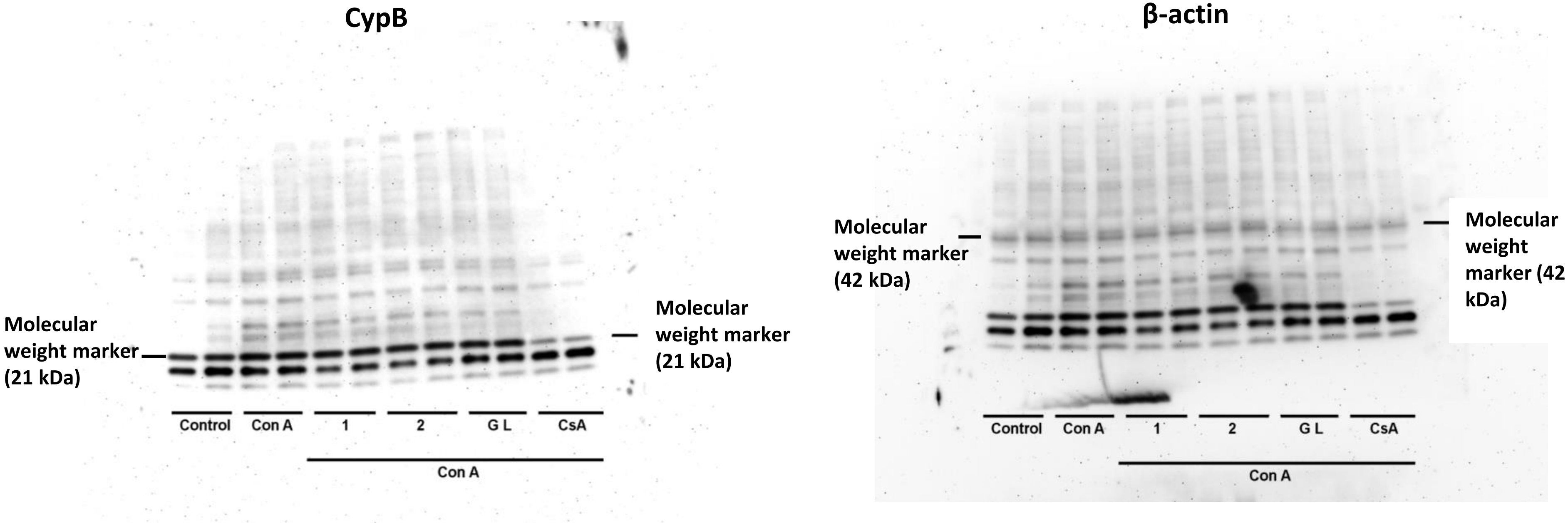


**Figure S4**. Uncropped images of western blot for CypB used in figure 3e. The immunoreactive bands were analysed using the Supersignal West Pico.

# Supplementary figure S5

**CypC**

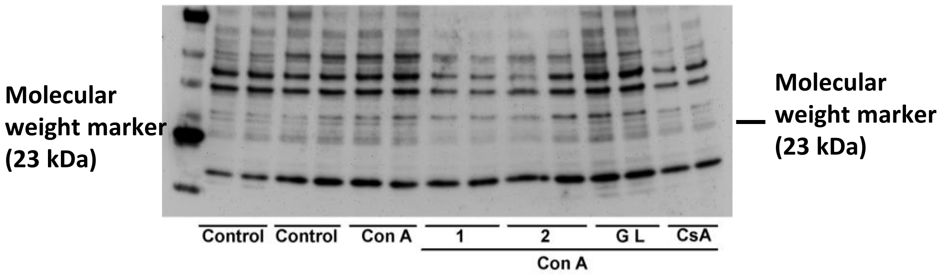


**β-actin**

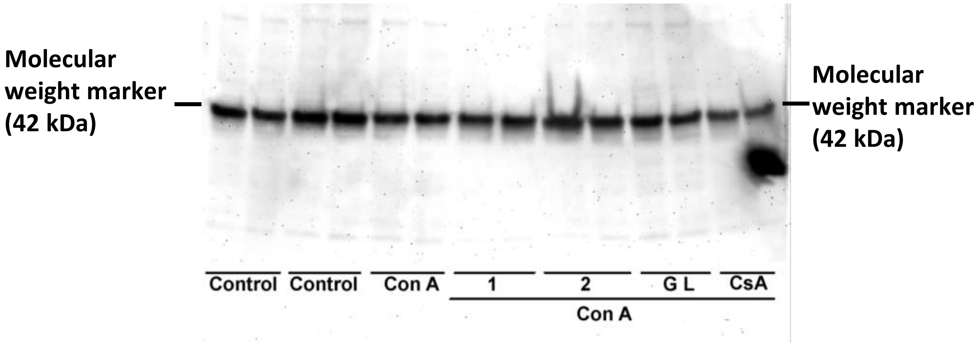


**Figure S5**. Uncropped images of western blot for CypC used in figure 3f. The immunoreactive bands were analysed using the Supersignal West Femto.
